# An Intelligent Multi-Task Supply Chain Model Based on Bio-Inspired Networks

**DOI:** 10.3390/biomimetics11020123

**Published:** 2026-02-06

**Authors:** Mehdi Khaleghi, Sobhan Sheykhivand, Nastaran Khaleghi, Sebelan Danishvar

**Affiliations:** 1 Department of Industrial Engineering, South Tehran Branch, Islamic Azad University, Tehran 15847-43311, Iran; 2 Department of Biomedical Engineering, University of Bonab, Bonab 55517-61167, Iran; 3 Faculty of Electrical and Computer Engineering, University of Zanjan, Zanjan 45371-38791, Iran; 4 College of Engineering, Design and Physical Sciences, Brunel University London, Uxbridge UB8 3PH, UK

**Keywords:** bio-inspired neural networks, sustainability, supply chain management, ensemble deep learning, DataCo, SupplyGraph, intelligent supply chain

## Abstract

Acknowledging recent breakthroughs in the context of deep bio-inspired neural networks, several architectural deep network options have been deployed to create intelligent systems. The foundations of convolutional neural networks are influenced by hierarchical processing in the visual cortex. The graph neural networks mimic the communication of biological neurons. Considering these two computation methods, a novel deep ensemble network is used to propose a bio-inspired deep graph network for creating an intelligent supply chain model. An automated smart supply chain helps to create a more agile, resilient and sustainable system. Improving the sustainability of the network plays a key role in the efficiency of the supply chain’s performance. The proposed bio-inspired Chebyshev ensemble graph network (Ch-EGN) is hybrid learning for creating an intelligent supply chain. The functionality of the proposed deep network is assessed on two different databases including SupplyGraph and DataCo for risk administration, enhancing supply chain sustainability, identifying hidden risks and increasing the supply chain’s transparency. An average accuracy of 98.95% is obtained using the proposed network for automatic delivery status prediction. The performance metrics regarding multi-class categorization scenarios of the intelligent supply chain confirm the efficiency of the proposed bio-inspired approach for sustainability and risk management.

## 1. Introduction

A compulsory stage of leading business is supply chain management. The interactivity of various representatives of suppliers, producers, marketers, purveyors and consumers plays a significant role in managing the supply chain. Regulations are made for the representatives with the aim of influencing the interactivity between each part for enhancing transportation and distribution services. Making a plan to optimize delivery, setting an appropriate balance of the demand and supply, documentation and identification of suppliers to prepare goods and services, and managing the return of products are the essential tasks in supply chain management [1].

There are some risks associated with the supply chain, and they should be detected. The procedure of detection, investigation and administration of these risks are the actions for risk mitigation in a supply chain. Also, risk management can be considered as the process of recognizing and managing the threats to the validity of services. There are many types of supply chain risks threatening companies and their service offer capacities. Some examples of these risks are the risks of different catastrophes, political and geographical vulnerabilities, malware, digital attacks and the suppliers’ financial failures [2]. An understanding of the risks is necessary for companies to manage the risks. It is an essential part of management to have an insight and manage risks. The COVID-19 epidemic is a tangible example of risks to supply chains [3,4]. Researchers developed a framework to handle the threatening risks of the supply chain during the epidemic [5]. A pragmatic risk administration method requires the identification of familiar and obscure risks, construction of a risk management system and execution of some techniques in order to reduce risks [6]. Extending the suppliers and making connections with them play an essential role in successful techniques for risk management in the supply network [7]. The investment in modern technologies is required for improving the strength, sustainability and discernibility of a supply chain [8].

Supply chain management includes different stages of planning, sourcing, manufacturing, delivery and returns. Prediction of the future production demand is considered to be the first stage of management. Identification of the suppliers is an important task of the second stage. Transportation of products and delivering them to the customers are performed in the third step. The fourth stage is processing product returns and refunds from the customers to mitigate costs [9]. A deep learning approach can be used to optimize the outcome of each stage. Deep learning has been utilized in supply chain studies in recent years. Deep networks are thoroughly bio-inspired architectures [10,11] and they extract fundamental concepts from brain activity. The convolutional networks mimic the hierarchical processing in the visual cortex and dendritic computation. These brain-inspired computational networks [12] have led to breakthroughs in artificial intelligence for understanding complex data. Novel deep learning methods add more complex biologic principles to the architecture of the neural networks [13]. This learning approach is fundamentally bio-inspired [14], drawing its core concept of a multilayered structure from the function of the human brain, using interconnected neurons to process data and learn complex patterns like the performance of biological brains. Like the brain learning from experience, deep learning approaches learn by adjusting connections on the basis of vast amounts of data. Multiple layers are used in deep learning, similar to how the visual cortex processes information. The hierarchical processing in this multilayered structure has the ability to learn highly complex representations. The basic building blocks are influenced by biological neurons, connecting in layers to learn deep features from the data. Artificial intelligence has helped to create intelligent supply chain models and bionic supply chains. An artificial intelligence-based supply chain [15] has the capability of catalyzing fundamental changes in business. A bionic supply chain [16] utilizes artificial intelligence methods for creating an AI-augmented workforce. Using these learning methods helps to create AI-based bionic supply chains [17]. A bionic supply chain works on the basis of a fusion strategy of human decision-making and artificial intelligence methods. It augments human capabilities with intelligent methods for enhancing the supply chain’s performance. The bionic supply chain does not remove the human workforce; it utilizes them and improves the performance of the intelligent supply chain methods. The bionic supply chain uses technological inventions to create an agile and resilient supply chain. This type of supply chain emphasizes collaboration between humans and machines to optimize supply chain operations. It leverages the power of artificial intelligence methods while acknowledging the key role of human decision-making. This collaborative method fosters a more adaptable supply chain [18].

Taking into consideration the necessity for creating intelligent supply chain models and bionic supply chains, an effective brain-inspired network is improved in this study for automated management tasks. The AI-based supply chain is an intelligent supply chain that offers end-to-end visibility and makes automated decisions for greater efficiency and customer satisfaction [19]. Regarding the connections among the product feature vectors, plant locations and allocated resources, it is possible to represent the supply chain as a graph. Graph representation is the compulsory prerequisite of graph-based deep learning. Graph-based deep learning is a strategy influenced by the connectivity between different brain regions during neuronal activities. It is a brain-inspired approach for creating intelligent systems [20]. An effective graph-based illustration of data samples is introduced in this study, and a brain-inspired graph network is introduced and developed to realize the delivery status for risk management. Moreover, product type classification and edge connection classification are performed for supply chain sustainability management. The suggested network creates an agile intelligent supply chain model that works on the basis of the bio-inspired convolutional neural network [21] and the brain-inspired graph neural networks [22,23].

The contributions presented in this article can be introduced as follows:

1. It suggests a hybrid brain-inspired graph network for extracting discriminative patterns and identifying different categories in a supply chain.
2. The proposed bio-inspired technique uses a graph illustration of the features recorded for products. The correlation between the characteristics of the products is employed to construct the connection graph, influenced by the functional connectivity in the brain. The functional connectivity refers to synchronized activity between different neuronal regions.
3. The characteristics related to the products are utilized directly as the nodes of the brain-based functional connectivity-inspired graph in the proposed method. This step is performed in order to decrease the calculation load in training phase.
4. The proposed ensemble intelligent supply chain model classifies the delivery status of the products, hence improving the performance of risk administration.
5. The proposed network architecture provides a framework for classification of 5 different product categories, 4 edge connections in terms of products with the same groups and 25 categories of products with the same plants. Hence, it develops the sustainability of the intelligent supply chain.
6. It uses a parallel network of brain-inspired Chebyshev-based graph convolution and bio-inspired 1-D convolution layers for creating an intelligent supply chain model.

The other sections of this paper are organized as follows. Section 2 unveils contemporary approaches of supply chain management using machine learning. In Section 3, the characteristics of the DataCo and SupplyGraph databases are provided. Also, the mathematical basis of brain-inspired graph convolution, Chebyshev graph convolution and graph attention networks are explained in this section. Section 4 describes the attributes and the structure of the bio-inspired ensemble network for different purposes. Some modeling targets are considered, including delivery status prediction, product classification and product connection classification for creating an intelligent multi-task supply chain model. Section 5 presents and extends the experimental results. The figures in this section substantiate the efficiency of the proposed bio-inspired ensemble network. Section 6 is allocated to the conclusions.

## 2. Related Works

The bio-inspired deep learning approach has the ability to analyze large amounts of data and extract learned features with trained parameters. Machine learning algorithms help to forecast time-based features and sales predictions. The selection of routes in a supply chain network can be obtained with machine learning approaches and deep learning algorithms to reduce transportation and delivery costs and plan an efficient route. Deep learning models and classification algorithms can be used for improving the model’s accuracy for the prediction of demand, detection of anomaly, optimization of logistics and sustainability of the supply chain [9]. Some studies applied deep learning in order to forecast real-time data related to the demand rate of production. Also, the objectives of deep learning have been detection of abnormal data, implementation of a predictive and sustainable maintenance plan, and promotion of decision-making [24,25,26]. Also, deep learning in supply chain management has been used to propose methods for supplier selection [27,28].

Pereira et al. in [27] proposed an analysis for the selection of suppliers. Their approach was designed corresponding to the CRITIC-GRA-3N machine learning approach proposed by Almeida et al. [29] in 2022. The method improved the selection of auto parts dealers in the city of Guaratingueta-SP. It was able to rank and select the suppliers in an efficient way. In the study by Ramjan Ali et al. [28], the authors identified a list of supplier selection benchmarks that apply to many organizations. The random forest classification method in conjunction with the RF-related feature extraction method was used in this study. The most critical criteria for supplier selection investigated in [28] are quality, material price, information sharing and on-time delivery. In another study by Yazdani et al. [30] in 2021, the interval-valued fuzzy neurosophic (IVFN) model was extended for the selection of suppliers for a dairy enterprise in Iran.

In some studies, a multi-phase approach based on deep learning has been proposed for allocating the orders in a supply chain [31,32]. In the study by Shidpour et al. [31] in 2023, a model was improved for developing the supply chain’s performance. The objectives in their study were allocating customer orders and selecting the suppliers. The corporate social responsibility scores were considered to acquire the ideal result for the model in [31]. The impact of deep learning-based transformation on decreasing the cost of transfers and transaction expenses in production management has been investigated by Li et al. [33]. Other examples of deep learning applications of this approach in supply chain management are deep modeling for transportation and conveyance issues during wars [34], a deep model of technology acceptance and the diffusion of innovation theory for production management in the supply chain [35].

Another applicable field of study for deep learning models is low-carbon methodologies for green supply chain administration [36]. In the study by Chun Fu et al. [36] in 2023, the impact of low-carbon activities in the construction industry was investigated. The framework in their study was based on the exploration of structural modeling based on least squares. The analysis of data was performed with the use of partial least squares in structural equation modeling (PLS-SEM). The analysis of supply chain relationships in [36] helped to propose plans in order to diminish carbon dioxide pollution emissions. The study had a positive effect on the surrounding environment. In the study by Niu et al. [37] in 2024, location choices for enterprises and allocated centers for distribution were involved in modeling. The proposed model in [37] reduced the cost with appropriate location choices for allocation centers and production plants.

Deep learning for risk management has been done in recent years for different objectives such as selection and segmentation of suppliers for risk prediction [38]. Estimation of suppliers’ responsiveness and improving the resilience and strength of the supply chain [39] are other examples of deep models’ application in risk management. The detection of disruption, fraud and anomaly are other objectives of deep models [40]. In the study by Sebastian Villa in 2022 [40], the authors made an exploration of the bullwhip effect in a supply chain. Some circumstances of horizontal competition between retailers were considered during the study. A mathematical model [40] was developed in a competitive system, while two behavioral explorations were considered to analyze the impact of supplier and customer characteristics on the decision of the retailers. The results of the study [40] demonstrate that competing for demand does not have an effect on how retailers expand their orders, whereas competing for supply influences the participants’ ordering decisions. Furthermore, the order variability decreased by up to 50% through modification of the supplier’s strategy for distribution in [40]. The retailers ignore the order cancellations of the customers according to this study [40].

Brain-inspired graph neural networks have been utilized by researchers in some studies related to supply chain management in recent years. Abushaega et al. in 2025 [41] considered graph learning in local centers of the supply chain network to optimize the global supply chain network. They used the concept of federated learning in order to improve the sustainability of the global network. The graph construction phase in their study was prominently based on the logistics and delivery services. The considered dataset was the Supply Chain Data dataset and was segmented into three subcategories: the distributor, the manufacturer and the supplier. The dataset characteristics in [41] pertain to the acquired raw material, the time of delivery and the costs of the supplier. Yuemei Sun in 2025 [42] used the concept of Graphsage network learning for risk prediction in financial transaction networks. An ATM security configuration for unusual activity detection was proposed in [43] by Kshirsagar et al., based on graph network learning. Input video files were collected from the UCF crime database and the DCSASS dataset in [43]. Abuse, shoplifting, arrest, burglary, explosion, assault, fighting, robbery, road accidents, shooting, arson and stealing were considered for investigation in this research [43]. Their approach demonstrates the reliability for real-time surveillance applications. Foroutan et al., in 2024 [44], performed graph learning for classification purposes and investigation of price fluctuation in crude oil markets. The dataset in their work [44] encompasses considerable markets and zones for a long period of about two decades.

In this paper, we propose a bio-inspired ensemble graph network for creating a multi-task intelligent supply chain model. The proposed intelligent model provides a framework for automatic risk administration and supply chain sustainability management. In the next section, we delineate the datasets’ characteristics and the mathematical foundation of the brain-inspired Chebyshev graph convolution kernel and graph attention layer.

## 3. Materials and Methods

In this section, the details of the two databases used in this study are explicated. The DataCo and the SupplyGraph databases are used in this study. Moreover, the mathematical basics of the brain-inspired graph convolution kernel and graph attention network will be elucidated to understand how the graph layers work.

### 3.1. Database Setting

Table 1 illustrates the details of the DataCo dataset. This dataset consists of 52 columns for 180,000 transactions of DataCo global company. Three types of transaction, days for shipment (scheduled), days for shipping, benefit per order, sales per customer, latitude, longitude, order item discount rate, order item discount, order item total and order profit per order are the characteristics for each data sample. The target labels for these data samples are the late delivery risk status. Table 2 presents the specifications of the target feature “late delivery risk,” which is defined as binary. In this feature, a value of 0 represents on-time delivery, and a value of 1 represents late delivery. The minimum value of this feature is 0, and the maximum is 1. This feature is used in predictive models for classifying the delivery status and helps simulate the likelihood of late delivery of goods or services.

SupplyGraph is the second dataset in this research. It consists of the features of products, companies and resources. It consists of nodes and edges labeled for connections between products for same groups and same plants, separately.

Fluctuations of and variations in delivery to the distributor, factory issue, production and sales orders for each day have been considered for this dataset. The nodes are available for product group, product sub-group and storage location. Edges are available for the connections between plants and connections between product groups. The characteristics of time-based features are in terms of units and weights. For the homogenous type of SupplyGraph database in Table 3, there are 40 products and there are 5 different categories for products and 25 categories of plants.

The edge indexes corresponding to different plants are available in this database. The connections according to different product categories are gathered in a file. The graph construction can be done corresponding to plants and product categories.

**Figure 1:**
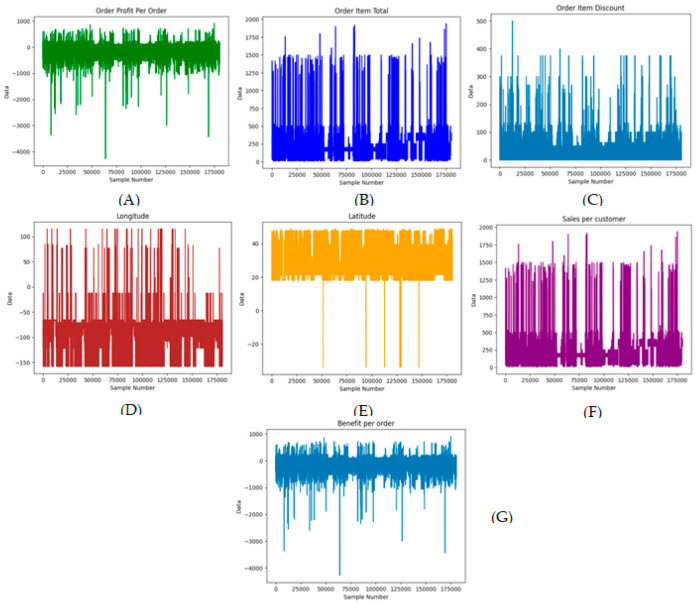
Characteristic plots for the Dataco dataset. (**A**) Order profit. (**B**) Order item. (**C**) Order discount. (**D**) Longitude of location. (**E**) Latitude of location. (**F**) Sales. (**G**) Benefit.

**Figure 2:**
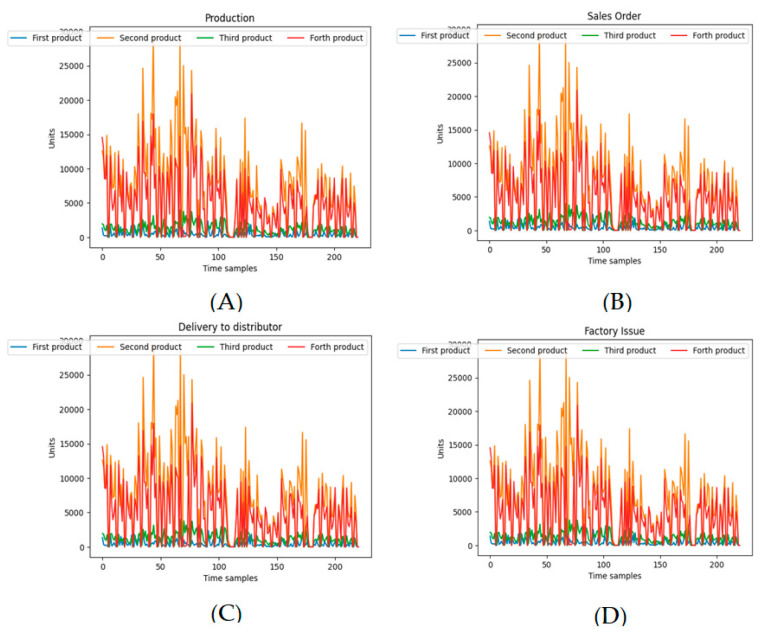
Characteristic time plot for 4 different products: (**A**) Production, (**B**) sales order, (**C**) delivery to distributor, and (**D**) factory issue.

Figure 1 illustrates DataCo’s characteristic signals for all transactions in the company. Figure 2 shows the fluctuations of production sales orders, delivery to the distributor and factory issue corresponding to with four products of the SupplyGraph.

Table 4 presents the edge connections in the homogenous SupplyGraph. It defines two edge types: “Plant” and “Product group,” both connecting products. The “Plant” edge type has 1647 connections, while the “Product group” edge type has 188 connections. This table illustrates the relationships between plants, product groups, and products in the supply network.

### 3.2. Graph Convolution

The research of Michaël Defferrard et al. [30] led to the popularization of graph signal processing (GSP). The mathematical functions in GSP take into account the attributes of the graph’s elements and also the structure of the graph. GSP is utilized to develop the convolution kernels of the graph domain, and this area of research exploits signal processing techniques like the Fourier transform and deploys them in graph representations. The use of the Fourier transform in GSP results in graph spectral filtering, which is named graph convolution [32]. Bio-inspired graph convolution refers to the development of graph convolutional networks that mimic natural biological mechanisms to process information. Bio-inspired meta-heuristic methods are frequently used for hyper-parameter optimization of graph convolution networks.

We explain the graph convolution layer in deep networks as described in [32]. Taking the graph structure into consideration, it is required to know the adjacency matrix and degree matrix according to the specific graph illustration. Here, $W\;\in \;{ℜ}^{\left(N\times N\right)}$ is considered as the adjacency matrix and $D\;\in \;{ℜ}^{\left(N\times N\right)}$ corresponds to the degree matrix. The calculation of the ***i***-th diagonal component of the degree matrix can be described by (1). The Laplacian matrix of the graph named ***L*** in the formula is acquired by (2).

(1) $$D_{ii}=\;\sum_{j}w_{ij}\;$$

(2) $$L=\;\;D-W\;\in \;{ℜ}^{\left(N\times N\right)}$$

The fundamental operations in the graph domain are computed in accordance with the eigenvectors of the graph Laplacian matrix denoted by ***U***. These vectors can be obtained via singular value decomposition (SVD) in (3).

(3) $$L=\;\;U\Lambda U^{T}$$

The columns of $U=\;[u_{0},\;\ldots ,\;u_{N-1}]\;\in \;{ℜ}^{\left(N\times N\right)}$ comprise the Fourier basis, and $\Lambda =\;diag\left([\lambda_{0},\;\ldots ,\;\lambda_{N-1}]\right)$ is a diagonal matrix. Calculation of the eigenvectors returns the Fourier basis in accordance with the graph. For a given signal $X\in \;{ℜ}^{N}$ denoting the stacked feature vectors on the graph nodes, its graph Fourier transform (GFT) via the obtained graph basis functions is expressed as shown in (4).

(4) $$\hat{X}\;=\;(U^{T})X$$

In Formula (4), $\hat{X}$ designates the converted signal in the frequency domain and is the answer corresponding to the graph Fourier transform. The formula above expresses that the inverse of the GFT can be obtained via the formula in (5). The filtered version of ***X*** by (***L***) can be written as shown in (6).

(5) $$X=U(U^{T})X=U\hat{X}\;$$

(6) Y = g(L) X

Using the following formulation in (7), it is obvious that the graph convolution of ***X*** with the vector of ***U****g*(***Λ***) is symmetrical to the kernel operation of (6). The g(Λ) in Formula (7) is expressed as shown in (8).

(7) $$\begin{array}{l} y=g(L)x=Ug(\Lambda )U^{T}x=U(g(\Lambda )).(U^{T}x)\\ =U(U^{T}(Ug(\Lambda ))).(U^{T}x)=x{*}_{g}(Ug(\Lambda )) \end{array}$$

(8) $$g(\Lambda )=\left[\begin{array}{l} g(\lambda_{0})\;\;\;\ldots \;\;\;\;\;\;\;\;\;\;0\\ \;\vdots \;\;\;\;\;\;\;\;\ddots \;\;\;\;\;\;\;\;\;\;\vdots \;\;\;\\ 0\;\;\;\;\;\;\;\;\ldots \;\;\;\;\;g(\lambda_{N-1}) \end{array}\right]$$

### 3.3. Chebyshev Graph Convolution

In this section, we explain a particular type of bio-inspired graph convolution entitled Chebyshev graph convolution, while replacing g(***L***) in (7) with the Chebyshev polynomial of ***L***. As we described earlier, the graph convolution of ***X*** with ***U****g*(***Λ***) can be calculated as shown in (9).

(9) $$y=g(L)x=g(U\Lambda U^{T})x=Ug(\Lambda )U^{T}x$$

The estimation of the g(***Λ***) is done via the *K*-order Chebyshev multinomials. The normalized version of ***Λ*** is utilized for approximation of the g(***Λ***) operation. The largest element among the diagonal entries of ***Λ*** is defined by *λMax*, and the normalized ***Λ*** is computed with the formula in (10). The ***I***_N_ in (10) is the ***N*** × ***N*** identity matrix, and the diagonal elements of $\tilde{\Lambda }$ lie in the interval of [−1, 1]. Approximation of g(***Λ***) based on the *K*-order Chebyshev polynomials framework as shown in Formula (10).

(10) $$\tilde{\Lambda }=\frac{2\Lambda }{\Lambda_{\max}}-I_{N}$$

(11) $$g(\Lambda )=\sum_{k=0}^{K-1}\theta_{k}T_{k}(\tilde{\Lambda })$$

In Formula (11), ***θ***k denotes the coefficient of the Chebyshev polynomials, and $T_{k}\left(\tilde{\Lambda }\right)$ can be acquired according to the following formulas in (12).

(12) $$\left\{\begin{array}{l} T_{0}\left(\tilde{\Lambda }\right)=1,\;T_{1}\left(\tilde{\Lambda }\right)=\tilde{\Lambda }\\ T_{k}\left(\tilde{\Lambda }\right)=2(\tilde{\Lambda })(T_{k-1}(\tilde{\Lambda }))-T_{k-2}(\tilde{\Lambda })\;\;\;,\;\;\;k\geq 2 \end{array}\right.$$

According to (12), the graph convolution kernel in (9) can be defined using (11) as illustrated in (13). In Formula (13), $\tilde{L}=2\frac{L}{\lambda_{\max}}-I_{N}$ is the normalized type of the Laplacian matrix.

(13) $$\begin{array}{l} y=U\;g(\Lambda )\;\;U^{T}x\\ =\sum_{k=0}^{K-1}U\left[\begin{array}{l} \theta_{k}T_{k}({\tilde{\lambda }}_{0})\;\;\;\ldots \;\;\;\;\;\;\;\;\;\;0\\ \;\vdots \;\;\;\;\;\;\;\;\;\;\;\ddots \;\;\;\;\;\;\;\;\;\;\;\vdots \;\;\;\\ 0\;\;\;\;\;\;\;\;\;\;\;\;\ldots \;\;\;\;\theta_{k}T_{k}({\tilde{\lambda }}_{N-1}) \end{array}\right]\;U^{T}x\\ =\sum_{k=0}^{K-1}\theta_{k}T_{k}(\tilde{L})x \end{array}$$

The expression of Chebyshev graph convolution in (13) indicates that it is symmetrical to the exploitation of the convolutional results of x with each part of the Chebyshev multinomial.

### 3.4. Graph Attention

Attention graph networks point out the restrictions of bio-inspired convolutional graph neural networks by enhancing optimizable self-attention procedures that allocate differing significance to different neighbors [45,46]. These are specialized deep learning architectures that integrate biological principles such as neural firing patterns into graph-based data processing. These networks use biological knowledge to weight connections between nodes, making them particularly effective for various regulatory tasks.

This section describes the formulation of the graph attention layer. A set of features is considered as the input of the graph attention layer as shown in (14). N and F designate the number of nodes and features, respectively.

(14) $$f\;=\;\{{\vec{f}}_{1},\;{\vec{f}}_{2},\;\ldots ,{\vec{f}}_{N}\}\;\;,\;\;{\vec{f}}_{i}\;\in \;R^{F}$$

A new set of node features would be created as the output of the graph attention layer.

(15) $$f\prime \;=\;\{\vec{f}\prime_{1}\;,\;\vec{f}\prime_{2},\;\ldots ,\vec{f}\prime_{N}\}\;\;,\;\;\vec{f}\prime_{i}\;\in \;R^{F\prime }$$

The weight matrix $W\in \;R^{F\prime \times F}$ is applied to every single node. The mechanism of self-attention is employed to calculate the attention coefficients:

(16) $$attention\;:\;R^{F\prime }\;\times \;R^{F\prime }\to \;Ra_{mn}\;=\;attention\;(W\;{\vec{f}}_{m}\;,\;\;W\;{\vec{f}}_{n})$$

A leaky rectified linear unit can be employed to calculate the normalized output, considering a nonlinear activation function.

(17) amn  = exp (LeakyReLU(w→T[concatenation(Wf→m , Wf→n))∑k∈Niexp  ( LeakyReLU(w→T[concatenation(Wf→m ,  Wf→k))

Considering the first-order neighboring nodes, the normalization process is performed across all choices of j using the softmax function:

(18) $${sa}_{mn}=\;soft\;\max_{n}\;(a_{mn})\;=\;\frac{\exp(a_{mn})}{\sum_{k\in N_{i}}\exp(e_{ik})}$$

The normalized attention coefficients are considered, and nonlinearity is imposed on the output.

(19) $$\vec{f}\prime_{m}\;=\;\Delta \;(\sum_{n\in N_{m}}sa_{mn}W{\vec{f}}_{n})$$

Concatenation of the features is required to construct the output.

(20) $$\vec{f}\prime_{m}\;=\;\overset{K}{\underset{k=1}{Concatenation}}\;\Delta \;(\sum_{n\in N_{m}}sa_{mn}^{k}W^{k}{\vec{f}}_{n})$$

For multi-head attention on the final layer of the network for prediction, averaging should be employed, and the final classification layer should be considered after the averaging step.

(21) $$\vec{f}\prime_{m}\;=\;\;\Delta \;(\sum_{k=1}^{K}\sum_{n\in N_{m}}sa_{mn}^{k}W^{k}{\vec{f}}_{n})$$

This technique enables the model to concentrate on more significant links and dependencies to improve the prediction performance. The negative side of the fact is the rising of the calculation cost and the incremental trend of complexity. In supply chain logical analysis, attention graph networks are specifically useful for anomaly detection and risk administration. Moreover, it is advantageous in cases where the capabilities of the model are used to rate essential connections and obtain more precise interpretations.

## 4. Proposed Bio-Inspired Method

**Figure 3:**
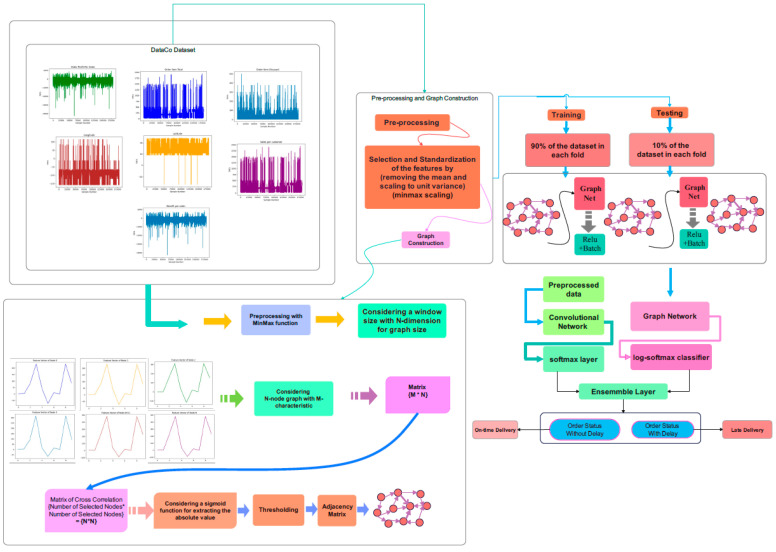
Schematic overview of the proposed method.

The graphical diagram of different stages in accordance with the proposed bio-inspired network is represented in Figure 3. The Dataco and SupplyGraph datasets are used in this study. As can be seen in this figure, after pre-processing of the data and graph design stage, the acquired graph would be applied to tune the parameters of the proposed bio-inspired Chebyshev ensemble graph network (Ch-EGN) during the training stage.

The network includes two distinct parts of deep networks. The brain-inspired graph-based section consists of four layers of Chebyshev convolution layers, and the bio-inspired convolutional part includes two sequential non-graph convolutional layers. The loss function of the ensemble network is the weighted summation of the parallel graph-based part and convolutional part of the network. The training phase of the Ch-EGN is performed with K-fold cross-validation.

### 4.1. Pre-Processing Stage

Dataco and SupplyGraph signals are considered in this study. The conversion of text-like features to integers is the first step of the pre-processing stage. The selection of features in order to clean the datasets is another step. The clean array of features is used and applied to the graph design phase.

### 4.2. Graph Construction

After pre-processing, the graph design stage is necessary to employ the acquired graph in the training phase of the proposed network architecture. The correlation of characteristic features in the transaction data in DataCo is required for graph embedding.

The target for training the proposed Ch-EGN is considered to be conversion of on-time delivery status and late delivery into zero and one, respectively. For the SupplyGraph database, the target is the product number for product classification, and is the plant number and product sub-group number for edge classification purposes.

**Figure 4:**
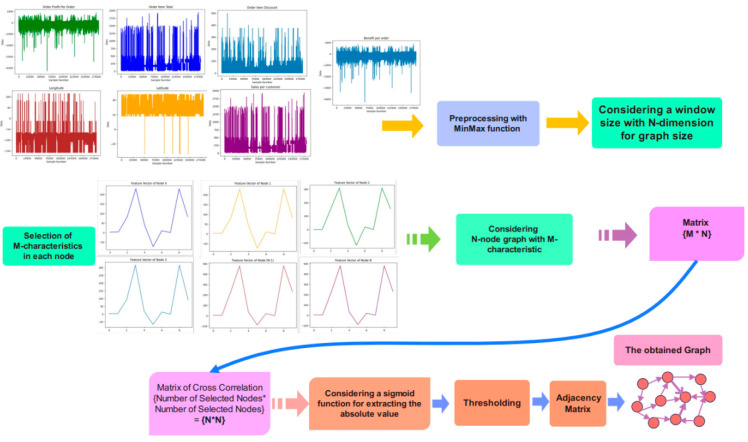
Graph construction stage for DataCo.

A sigmoid is utilized for computing the absolute value of the cross-correlation matrix. Also, a threshold level is considered in order to remove some non-zero elements of the output array. The adjacency matrix is the output of the sigmoid function and thresholding stage according to the simplified graphical representation of the graph design stage in Figure 4.

### 4.3. Proposed Bio-Inspired Ch-EGN Architecture

**Figure 5:**
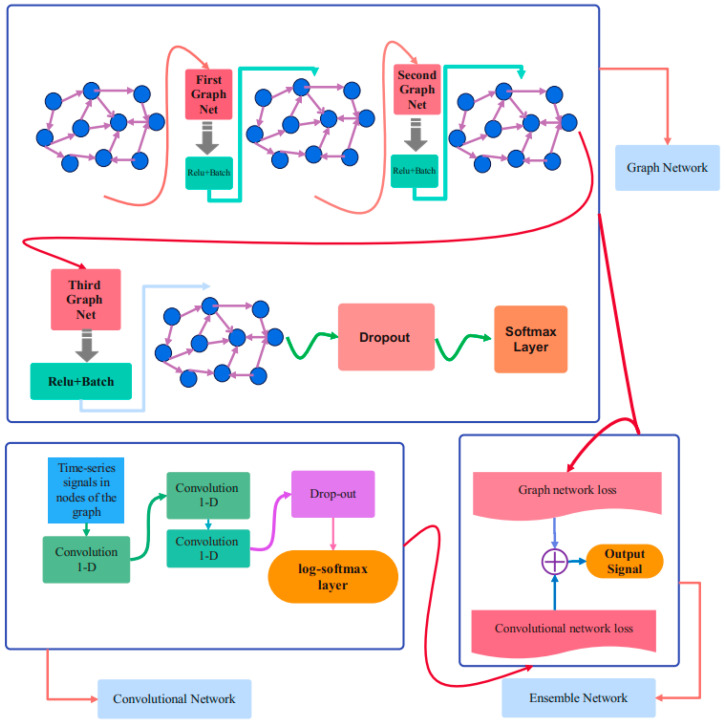
The detailed architecture of the proposed Ch-EGN.

Figure 5 delineates the detailed graphical representation of the proposed bio-inspired network architecture. As this figure shows, our proposed geometric Ch-EGN contains four layers of graph convolution. As specified by this figure, in every Chebyshev convolutional layer, the first step is the estimation of the Chebyshev convolution of the input graph via the graph Laplacian. The next layer is the activation layer. Also, batch normalization is utilized in the output of each layer to normalize the input to the next layer.

The output of the pre-processing stage is imposed to the parallel convolutional part of the ensemble bio-inspired network. The loss function is the ensemble of two loss functions of the parallel parts of the Ch-EGN network. After the log-softmax layers in parallel networks, the obtained signal is classified according to the target vector.

Batch normalization makes the network stable throughout the training procedure, and the convergence of the network would happen more quickly. The normalization is allocated to each graph convolution layer. After four layers of Chebyshev convolution and two parallel convolution layers, the extracted feature array is acquired, which is compatible with the size of the target vector.

The details and characteristics of the proposed architecture are explained in Table 5 and Table 6. Table 5 is related to the details of first part of the Ch-EGN. Table 6 is the attributes of layers matching the convolutional part of the network. Also, it shows the kernel size for different layers, the size of strides in the layers, the number of kernels used for each layer and the total number of weights to be trained during the training procedure.

The target vector for delivery status prediction in DataCo is a two-class vector. The target vector for SupplyGraph is 5 for product group classification, 4 for product sub-group classification and 25 for plant classification. Table 7 demonstrates the weight parameters of the edge classification part of the network for classifying different categories of the edge connections of the graph.

The 1-D convolutional layer assumes a fixed ordering of elements where the distance between neighboring units is constant. It performs a sliding window operation. It computes the dot product between a learnable kernel and a local segment of the input. The filter is translation-invariant, meaning that the same weights are applied across different positions in the sequence. It excels at capturing local patterns in ordered sequences, whereas the Chebyshev graph convolution handles irregular structures where the nodes have a variable number of neighbors with no fixed inherent ordering. The graph convolution has been designed for non-Euclidean data illustrated as graphs. The distance in the irregular structure of a graph is defined by connectivity edges rather than the spatial patterns of neighboring nodes. This type of convolution utilizes a spectral method estimated by Chebyshev polynomials. Instead of a sliding window, it operates on the graph Laplacian to approximate a filter in the spectral domain, as defined in Section 3. This approximation leads to the capture of global structures and long-distance dependencies across the graph. The 1-D convolution layer assumes a linear connectivity. However, the Chebyshev graph convolution adapts to complex relationships defined by a custom adjacency matrix.

### 4.4. Training and Evaluation of the Proposed Ch-EGN

In the training procedure, the generated input and target samples are utilized to tune the parameters of the suggested Ch-EGN to the Dataco and SupplyGraph datasets. We implement a 10-fold cross validation. 

**Figure 6:**
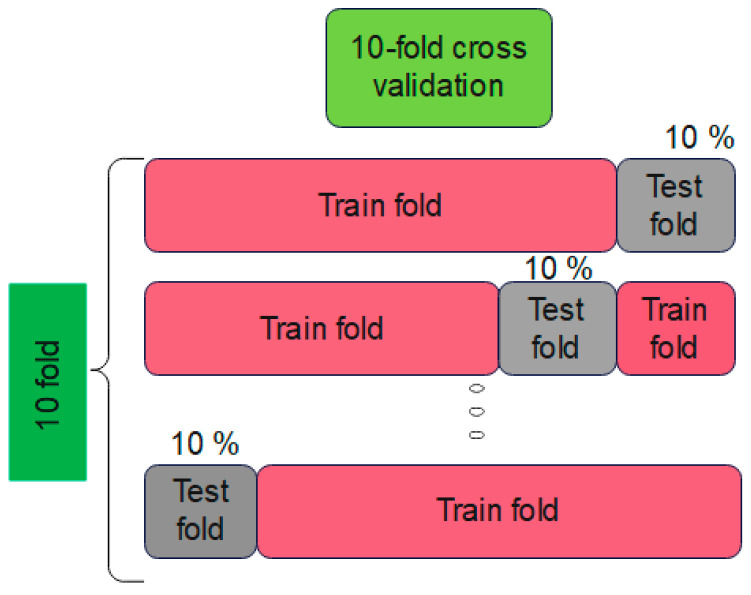
K-fold cross-validation stage.

After training and tuning the variables and parameters of the Chebyshev graph convolution network and the parallel convolutional network, the testing phase is performed. The training of the proposed Ch-EGN is performed according to the parameters in Table 5, Table 6 and Table 7. The optimal weights are obtained and summarized in this table. Cross-validation is selected for the validation procedure. A schematic view of this phase is indicated in Figure 6.

A 10-fold cross-validation is fulfilled in accordance with Figure 6 using the training samples. The test stage can predict the delivery status of Dataco and the classification purposes of SupplyGraph based on the calculated weights of the training stage. The pseudo-code in Algorithm 1 explains the details of the proposed Ch-EGN. Table 8 shows the training search area and the optimal parameters for each scope.
**Algorithm 1. Bio-inspired Chebyshev ensemble graph network (Ch-EGN)**Input: (1) Characteristic vectors ***X***; (2) A threshold level for adjacency matrix; (3) Chebyshev polynomial orders for each layer K_1_, K_2_, K_3_, K_4_; (4) Labeled train and test samples ***Xtrain*** and ***Xtest***; (5) α coefficient in ensemble cost function. Output: Class labels for X_test_ Initialize the model parameters. Repeat according to the 10-fold cross-validation: 1: Determine the correlation co-efficient of the of ***X*** in ***Xtrain***. 2: Calculate the adjacency matrix ***W*** by using the sigmoid function for the result of Step 1. 3: Determination of the normalized Laplacian matrix $\hat{\Lambda }$. 4: Calculate the multinomials in accordance with the layer. 5: Extract the output of the four Chebyshev graph convolutional layers considering *K*1, and using *K*2, *K*3 and *K*4 and the sequential activation layers. 6: Calculate the output of the dropout layer. 7: Calculate the output of the parallel simple convolutional layers. 8: Optimize the weights of the ensemble layers using appropriate loss function such as cross-entropy. 9: Update the weights of the layers using the total ensemble cost function:
$\begin{array}{l} \left\{\begin{array}{l} Loss_{Cross-Entropy}(t\arg et,{output}_{1})\;=\;-\;\frac{1}{n}\;\sum_{i=1}^{n}\left(t\arg et_{i}.{\log\;output}_{1i}+({output}_{1i}-t\arg et_{i}).\log(t\arg et_{i}-{real}_{i})\right)\\ Loss_{Cross-Entropy}(t\arg et,{output}_{2})\;=\;-\;\frac{1}{n}\;\sum_{i=1}^{n}(t\arg et_{i}.\log\;{output}_{2i}+({output}_{2i}-t\arg et_{i}).\log(t\arg et_{i}-real_{i}))\;\; \end{array}\right.\\ Loss_{Total}=\;Loss_{Cross-Entropy}(t\arg et,{output}_{1})\;+\alpha *\;Loss_{Cross-Entropy}(t\arg et,{output}_{2}\;) \end{array}$ 10: Obtain the predictions for the embedded graphs in accordance with ***Xtest*** using the trained Ch-EGN. Stop specifications: A maximum number of trials or acceptable accuracy.

**Figure 7:**
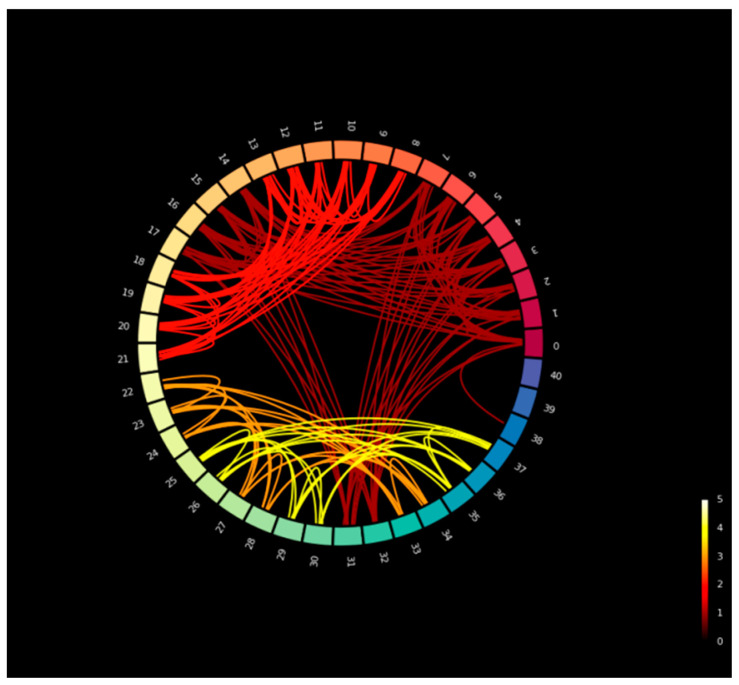
Circular connectivity between products in terms of product groups’ edge indexes.

**Figure 8:**
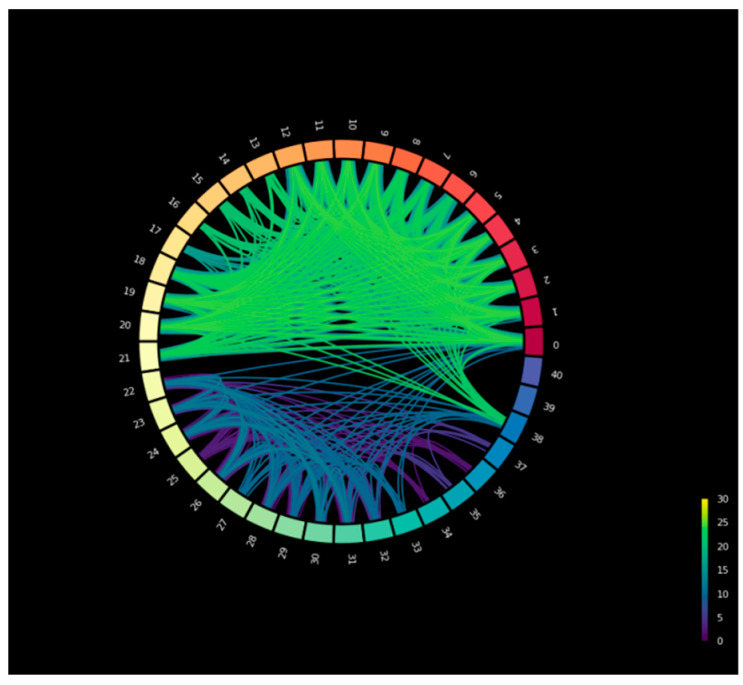
Circular connectivity between products in terms of plants’ edge indexes.

Figure 7 illustrates the circular connectivity of the edges in the SupplyGraph dataset with similar product groups. The edge connections are the relations between products in terms of same product types. Figure 8 is the circular connectivity between nodes of the SupplyGraph dataset considering the edge connections with similar plant locations. There are 188 edge connections in Figure 7 and 1646 edge connectivity in Figure 8 in terms of the similarity of the plant locations.

## 5. Results and Discussion

In this section, the results obtained through an analysis of the proposed bio-inspired Ch-EGN are presented. Our configuration is executed on a laptop with 16 GB RAM, a 2.8 GHz Core i7 CPU and a GeForce GTX 1050 GPU. The implementation of the proposed network was performed using the Google Colab Pro platform.

**Figure 9:**
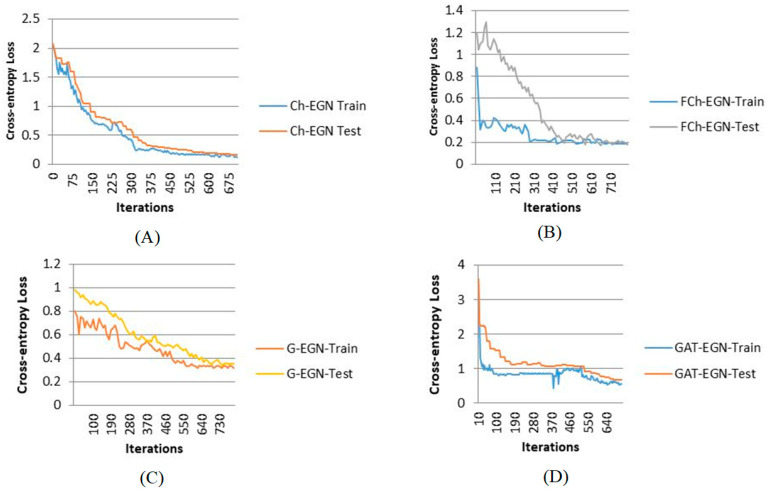
Loss plots for training on DataCo: (**A**) Ch-EGN; (**B**) FCh-EGN; (**C**) G-EGN; (**D**) GAT-EGN.

**Figure 10:**
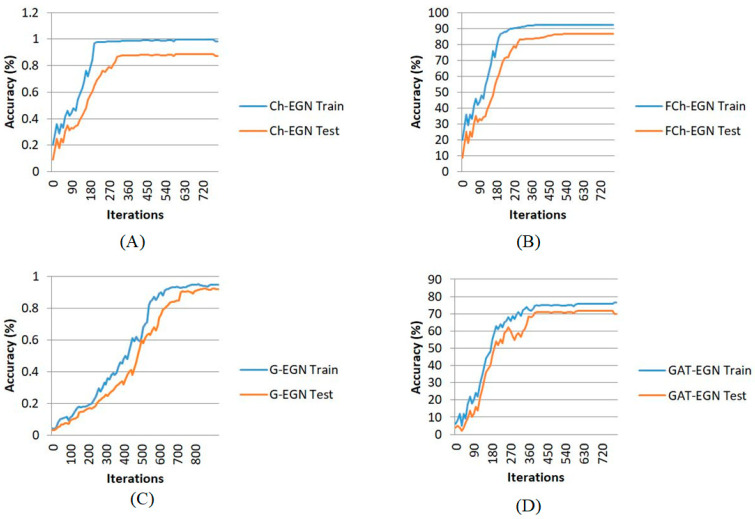
Accuracy plots for training the on DataCo.: ((**A**) Ch-EGN; (**B**) FCh-EGN; (**C**) G-EGN; (**D**) GAT-EGN).

Figure 9 and Figure 10 show the performance of the proposed Ch-EGN for DataCo, based on the loss functions in accordance with the Chebyshev graph convolution segment and the parallel convolutional network. Regarding these figures, the Adam optimizer with an optimal learning rate of 0.0001 and an optimum weight decay of 4 × 10^−4^ was used, taking into consideration the cross-entropy for the first segment of the network and the total loss corresponding to the pseudocode for the ensemble segment of the proposed network. This figure illustrates the loss plots for the Ch-EGN, fuzzy Ch-EGN, G-EGN and GAT-EGN. The fuzzy version needs more iterations in order to converge. The graph convolutional and graph attentional methods have weak performance in comparison with the Chebyshev convolutional network. Three layers of graph convolution networks are considered for the G-EGN, and the GAT includes three sequential layers of graph attention. As can be seen, we consider more than 700 iterations for all methods, considering a 10-fold cross-validation.

**Figure 11:**
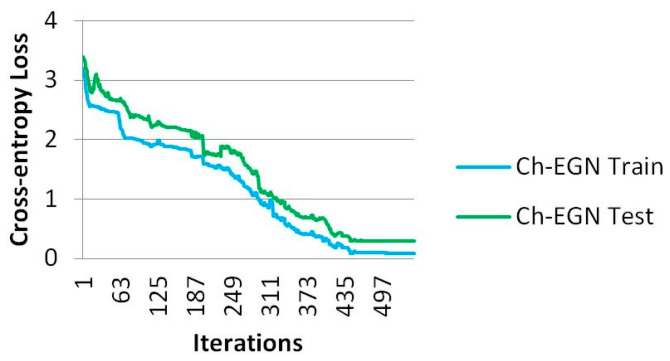
Loss plot for training the proposed method for the SupplyGraph dataset.

**Figure 12:**
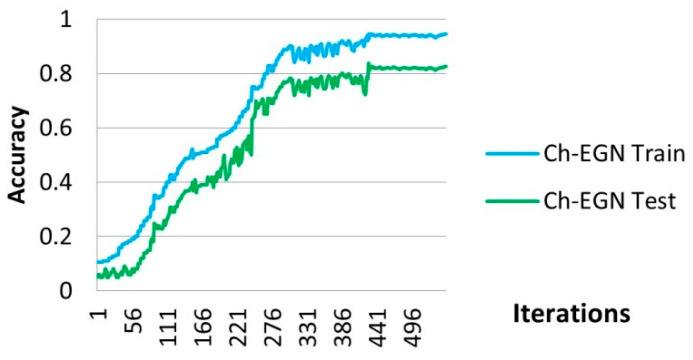
Accuracy plot for training the proposed method for the SupplyGraph dataset.

Figure 11 and Figure 12 demonstrate the performance of the Ch-EGN for SupplyGraph. The number of repetitions necessary for the convergence of the proposed method with the aim of product type classification with this dataset is equal to 500. The SupplyGraph dataset requires more than 500 iterations in order to converge.

Table 9 reports the performance metrics considering the DataCo dataset for prediction of the delivery status for different methods. This table shows the on-time delivery and late delivery status predictions’ accuracy. It also demonstrates the precision, F1-score and recall, considering various orders for the Chebyshev polynomial convolution layer and the FCh-EGN, G-EGN and GAT-EGN methods.

**Figure 13:**
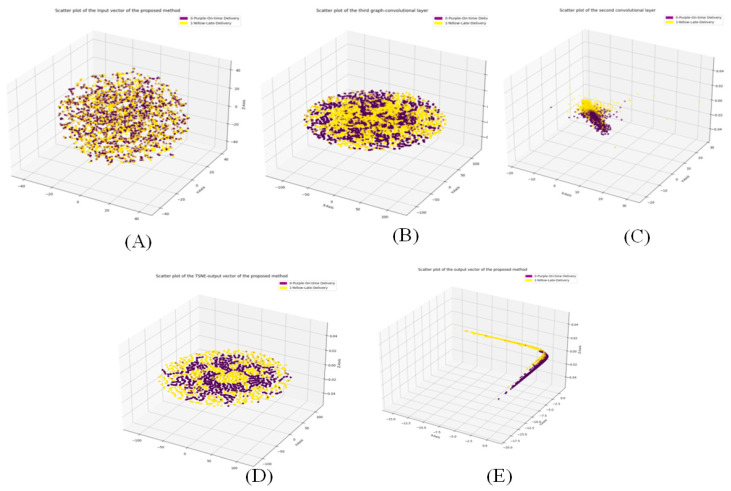
Three-dimensional TSNE plots for DataCo. (**A**) Input vector, (**B**) third graph layer, (**C**) second convolution layer, (**D**) output layer and (**E**) output of the softmax layer.

**Figure 14:**
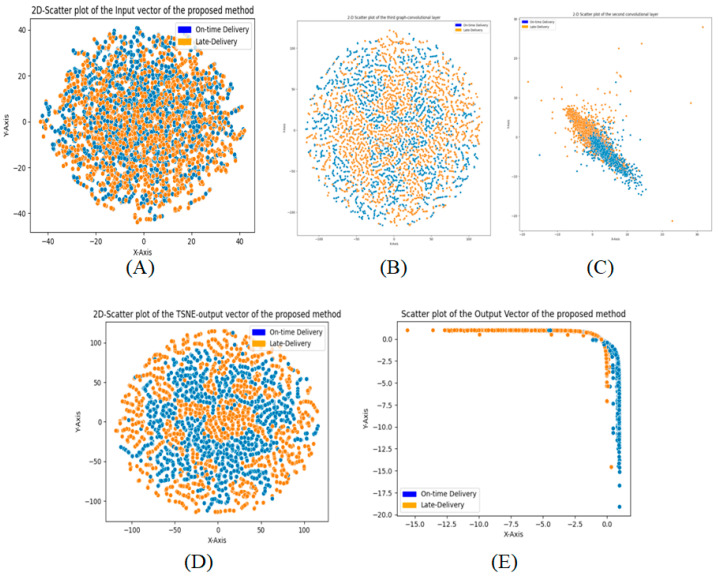
Two-dimensional TSNE plots for DataCo. (**A**) Input vector, (**B**) third graph layer, (**C**) second convolution layer, (**D**) output layer and (**E**) output of the softmax layer.

Figure 13 and Figure 14 illustrate the three-dimensional and two-dimensional T-SNE plots for different layers of the proposed Ch-EGN in order to demonstrate the procedure of the classification and a tangible view of the stages of classification considering the proposed Ch-EGN with the DataCo dataset.

**Figure 15:**
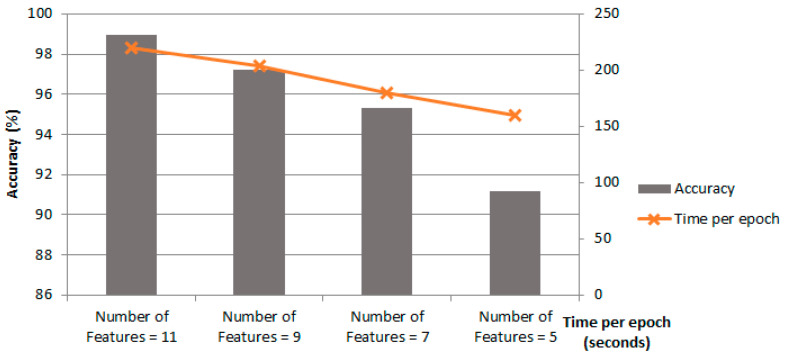
Test accuracy and time of training per epoch with different sets of features for DataCo graph construction.

Figure 15 shows the performance metrics of the proposed Ch-EGN regarding different sets of features for the DataCo database. The comparison shows a decreasing trend for delivery status prediction and time per epoch. As can be seen, decreasing the number of features affects the speed of processing positively. Although it will reduce the complexity and computational burden, it affects the performance’s accuracy negatively.

Table 10 is the report for product classification in the case of considering the SupplyGraph dataset. The classification accuracies for different types of product S, P, A, M and E can be seen in this table. The results of the proposed Ch-EGN together with those of the G-EGN, FCh-EGN and GAT-EGN are available in Table 10 for the SupplyGraph dataset. Moreover, this table presents the category-specific evaluation metrics thoroughly for all of these networks. As can be seen in Table 10, the proposed Ch-EGN surpasses the other methods.

Table 11 shows the detailed results for classification of the product type, product relation classification in terms of products and product connection classification in terms of plant similarity. There are five different categories of product types. There are 4 different groups of relations based on the product relations and 25 different groups of connections for similar plants. This table confirms the good performance of the proposed method for node and edge classification in comparison with other methods. The proposed Ch-EGN outperforms other methods for classification of the nodes and edges of the SupplyGraph database.

Table 12 shows the performance metrics of the proposed method in comparison with the other novel and traditional methods. As can be seen, our proposed geometric ensemble network outperforms the other conventional methods.

**Figure 16:**
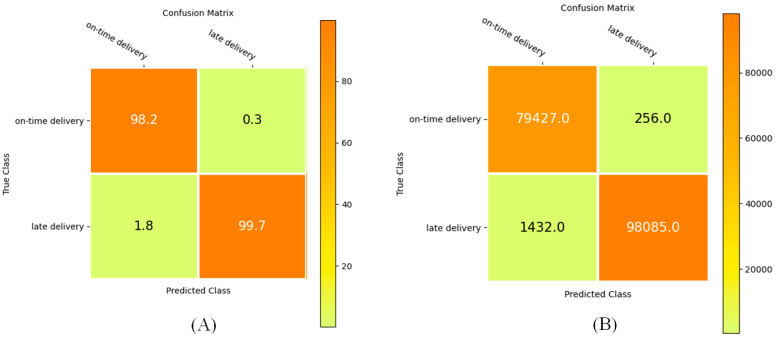
The confusion matrix for delivery status prediction. (**A**) Percentage. (**B**) Number.

**Figure 17:**
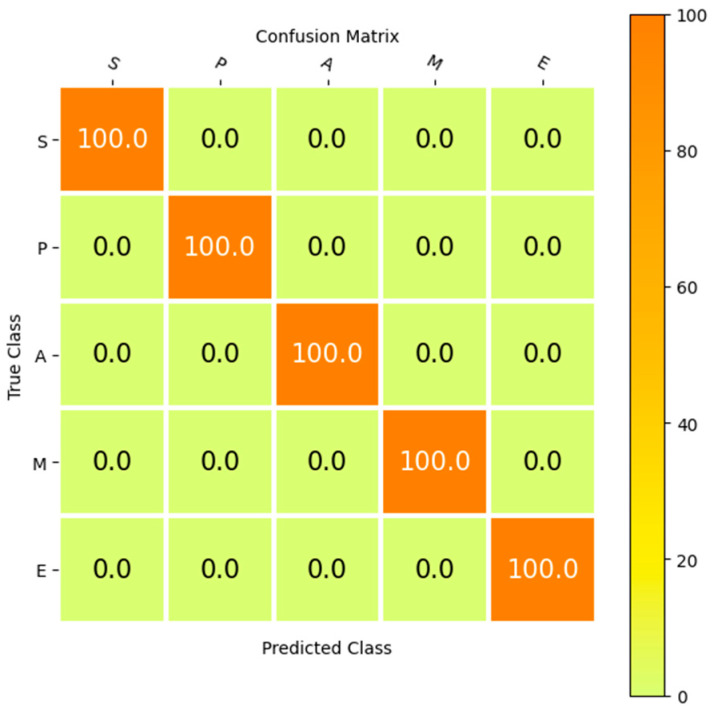
Product classification in the SupplyGraph database.

The confusion matrix is a valuable way of confirming the efficiency of the proposed method. Figure 16 shows the performance of the proposed Ch-EGN considering the DataCo dataset. Figure 17 is the confusion matrix for the classification of product types in the SupplyGraph dataset considering our proposed Ch-EGN.

**Figure 18:**
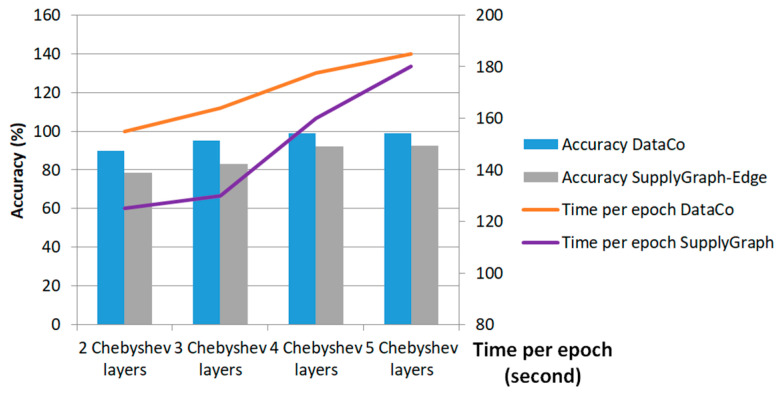
F1-score and time of training per epoch with different numbers of graph convolution layers.

To investigate the effect of different parameters on the optimality of the performance, we executed an extended experiment. In order to evaluate the effect of alternating the number of sequential Chebyshev filters, a series of training procedures were performed for different numbers of sequential Chebyshev multinomials. Figure 18 showcases the results of tuning for two, three, four and five sequential Chebyshev layers. Setting the sequential layers to more than four in this case study does not improve the performance, but it affects the computational complexity. This figure showcases the incremental direction of the training time per iteration epoch of the proposed Ch-EGN.

**Figure 19:**
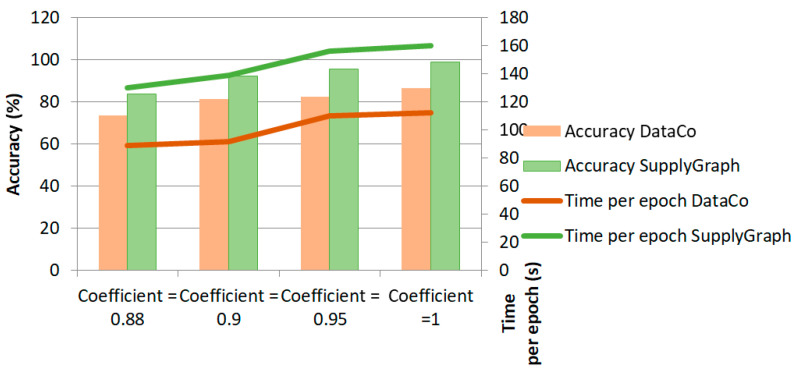
F1-score and time of training per epoch with different ensemble coefficients in the cost function.

Figure 19 is the comparison outcome of different altering coefficients α of the ensemble loss function in Algorithm 1. This column chart outlines that adjusting the coefficient equal to 0.9 will optimize the accuracy and that this is the most effective one, taking into consideration the converging time.

**Figure 20:**
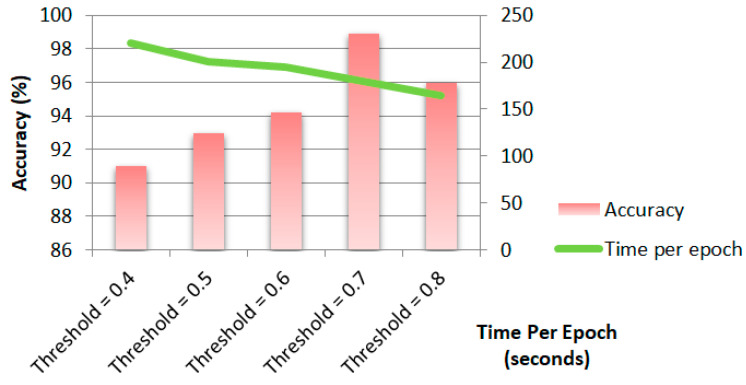
Test accuracy and time of training per epoch with different threshold levels for DataCo graph construction.

Figure 20 shows the performance metrics of the proposed Ch-EGN regarding different threshold levels. The comparison shows that considering a threshold level for graph construction equal to 0.7 results in a compromise between accuracy and computational burden.

**Figure 21:**
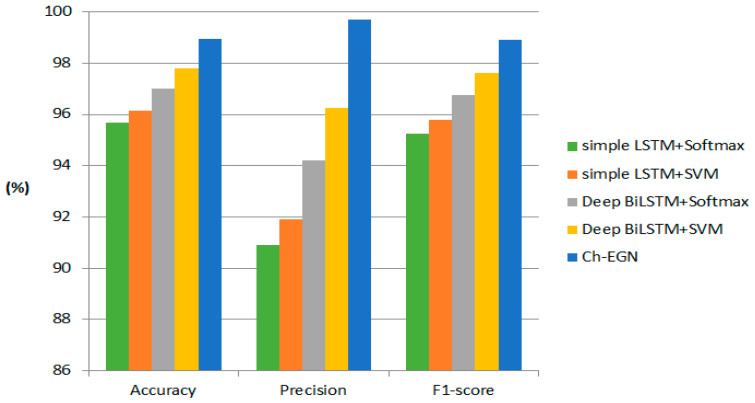
The comparison with other novel methods for DataCo in terms of accuracy, precision and F1-score.

Figure 21 shows the performance metrics of the proposed Ch-EGN in comparison with the other novel methods. The comparison shows the results of delivery status prediction using simple LSTM and BiLSTM methods along with softmax and SVM classifiers. As can be seen, our proposed geometric ensemble network outperforms the other methods provided in [50].

**Figure 22:**
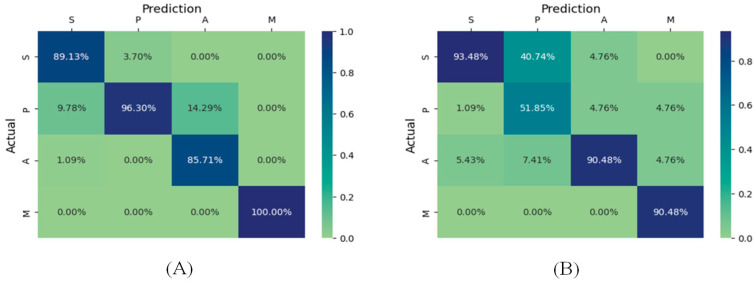
The confusion matrix for edge classification for product group connections. (**A**) Delivery to the distributor; (**B**) factory issue.

**Figure 23:**
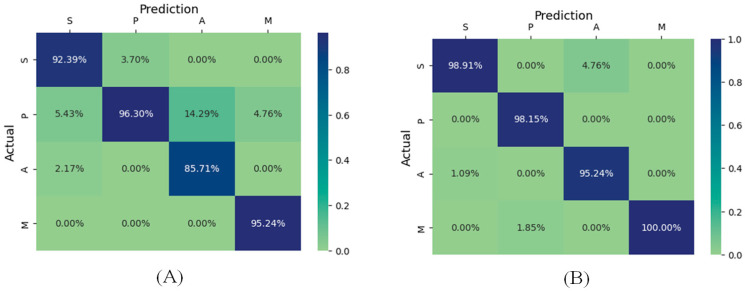
The confusion matrix for edge classification for product group connections. (**A**) Sales orders; (**B**) production.

Figure 22 and Figure 23 are the confusion matrixes for edge classification for the SupplyGraph database considering the proposed Ch-EGN. Figure 22A considers delivery to the distributor for the nodes. Factory issue has been considered for calculating the right-hand confusion matrix in Figure 22B. Figure 23A considers the sales orders for calculating the confusion matrix. The time-series of production has been considered for the confusion matrix in Figure 23B. All of these performance metrics confirm the efficiency of the proposed method.

**Figure 24:**
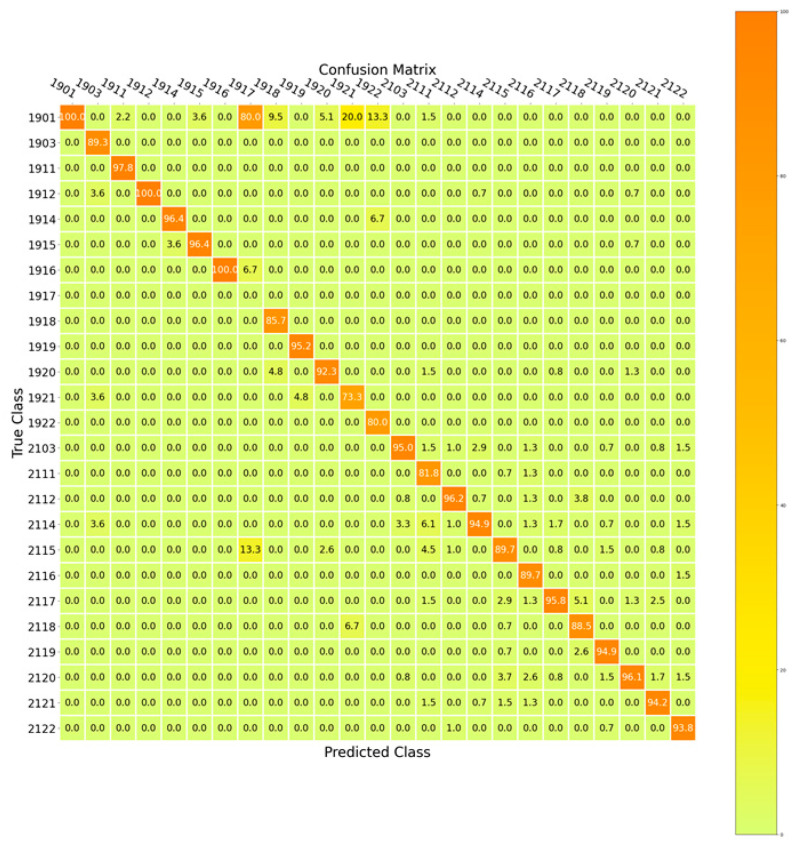
The confusion matrix for edge connections in terms of plant labels.

Figure 24 showcases the confusion matrix for edge classification of the SupplyGraph dataset considering the edge connections for similar plant locations for different types of products. This figure confirms the efficiency of our proposed intelligent multi-task supply chain model.

Recent studies demonstrate that intelligent supply chain systems increasingly rely on the integration of artificial intelligence and data-driven decision-making frameworks. Blockchain-enabled architectures have proven effective in enhancing transparency, traceability and waste reduction in food and healthcare supply chains, providing a reliable foundation for decentralized coordination [51]. Machine learning and deep learning techniques have been widely applied to complex engineering systems, including aircraft design, smart energy networks and large-scale optimization problems, showing superior performance in handling uncertainty and multi-objective constraints [52,53]. At the same time, the expansion of digital systems highlights the need for resilient and human-aware intelligent frameworks that account for behavioral and societal impacts [54].

In healthcare-related supply chains, artificial intelligence has enabled significant improvements in diagnosis, treatment planning and service management. Multi-task and multi-modal learning approaches have demonstrated strong capabilities in processing heterogeneous clinical and logistical data, particularly in uncertainty-sensitive environments [55,56]. Hybrid decision-making and multi-criteria evaluation methods further support the structured prioritization of AI-driven strategies in healthcare and service-oriented supply chains [57,58]. Intelligent optimization techniques have also been extensively employed in energy-aware infrastructures, where stochastic and interval-based models improve resilience and operational efficiency in smart grids and micro-energy networks [59,60,61,62]. These findings emphasize the importance of adaptive and bio-inspired optimization mechanisms in interconnected supply chain systems.

Advances in intelligent diagnostic systems and medical image analysis confirm the effectiveness of deep learning, semi-supervised and generative models in high-dimensional and data-limited environments [63,64,65,66]. In parallel, progress in causal inference, fair learning and large language models has expanded the scope of intelligent decision-support systems toward more robust and generalizable solutions [67,68,69,70,71]. Human-centered studies in spatial behavior, architectural intelligence and adaptive environments further highlight the role of contextual and behavioral factors in intelligent system design [72,73,74]. Moreover, recent research in demand response, energy coordination and sustainable infrastructure demonstrates the effectiveness of intelligent coordination mechanisms in large-scale interconnected systems [75].

Finally, uncertainty-aware learning paradigms, including fuzzy neural networks and advanced feature extraction methods, have shown high reliability in medical and signal-based intelligent systems, reinforcing the value of bio-inspired and adaptive learning structures for multi-task environments [76,77,78,79]. Collectively, these studies provide a solid foundation for the proposed intelligent multi-task supply chain model, which integrates bio-inspired networks with learning-based optimization to achieve scalable, adaptive and resilient supply chain intelligence [80].

## 6. Conclusions

In this paper, a novel bio-inspired deep ensemble architecture is proposed to create a smart supply chain model. The proposed model solves the problem of predicting the delivery status for risk management in a supply chain. In addition, it is an intelligent model proposed for strengthening the sustainability of the supply chain. The proposed model architecture is used for testing the sustainability of the SupplyGraph database and it is utilized for risk management with the DataCo supply chain dataset.

The connectivity between the nodes of the proposed ensemble method is influenced by the brain functional connectivity measurement between different brain regions during neuronal activities. This connectivity constructs the supply chain graph using the hidden states of supply chain characteristic vectors. The bio-inspired ensemble approach employs the supply chain graph for different scenarios of node and edge classification. The proposed ensemble brain-inspired deep network is a novel approach for supply chain automation. It facilitates risk management in a supply chain along with strengthening the supply chain’s sustainability. The efficiency of the proposed bio-inspired method for building an intelligent supply chain is delineated via the exploratory outcomes on the DataCo and SupplyGraph datasets. The proposed bio-inspired Ch-EGN creates an agile and transparent multi-task sustainable supply chain.

## Figures and Tables

**Table 1:** DataCo specifications.

DataCo	Feature	Format	Min	Max	Description
1	Type	Word	0	2	Kind of transaction
2	Real number of shipping days	Digit	0	6	Actual working days for shipping activities of the purchased product
3	Scheduled number for shipment days	Digit	1	4	Number of days for scheduled transportation of the purchased product
4	Gain per order	Numeral	−613.77	186.23	Gaining per order
5	Sales per customer	Numeral	27.04	399.98	Total sales per customer
6	Latitude	Numeral	18	44	Latitude according to storage location
7	Longitude	Numeral	−120	−66	Longitude according to storage location
8	Order item discount	Numeral	0	99.99	The discount value of the order item
9	Order item discount rate	Numeral	0	0.18	Discount percentage value of the order item
10	Total order item	Numeral	9.37	479.95	Total amount per order
11	Order profit rate per order	Numeral	−613	153	Profit rate per order

**Table 2:** Target feature specifications.

DataCo	Target Feature	Format	Min	Max	Target Description
1	Late delivery risk	Binary	0	1	1 for late delivery, 0 for on-time delivery

**Table 3:** SupplyGraph dataset’s characteristics.

DataCo	Feature	Duration	Number of Products
1	Temporal data—delivery to distributor	9 August 2023–1 January 2023	40
2	Temporal data—factory issue	9 August 2023–1 January 2023	40
3	Temporal data—production	9 August 2023–1 January 2023	40
4	Temporal data—sales order	9 August 2023–1 January 2023	40

**Table 4:** Edge connections in the homogenous SupplyGraph.

DataCo	Edge Type	Nodes	Number of Connections
1	Plant	Products	1647
2	Product group	Products	188

**Table 5:** Layers of the graph section of the proposed method.

Layers			DataCo			SupplyGraph		
Layer	Layer Name	Activation Function	Dimension of Weight Array	Dimension of Bias	Number of Parameters	Dimension of Weight Array	Dimension of Bias	Number of Parameters
1	Chebyshev convolution layer		[1, 10, 10]	[10]	110	[1, 220, 220]	[220]	48,620
2	Activation Layer	Relu						
3	Batch normalization		[10]	[10]	20	[220]	[220]	440
4	Chebyshev convolution layer		[1, 10, 5]	[5]	55	[1, 220, 100]	[100]	22,100
5	Activation Layer	Relu						
6	Batch normalization		[5]	[5]	10	[100]	[100]	200
7	Chebyshev convolution layer		[1, 5, 2]	[2]	12	[1, 100, 20]	[20]	2020
8	Activation layer	Relu						
9	Batch normalization		[2]	[2]	4	[20]	[20]	40
10	Chebyshev convolution layer		[1, 2, 2]	[2]	6	[1, 20, 5]	[5]	105
11	Activation layer	Relu						
12	Batch normalization		[2]	[2]	4	[5]	[5]	10

**Table 6:** Details of the convolutional part of the proposed method.

Data	Layer	Layer Name	Activation Function	Output Dimension	Size of Kernel	Stride Shape	Number of Kernels	Number of Weights
DataCo	1	Convolution 1-D	LeakyReLU(alpha = 0.1)	(10, 10, 5)	1 × 5	1 × 1	10	510
	2	Convolution 1-D	LeakyReLU(alpha = 0.1)	(2, 10, 5)	1 × 5	1 × 1	2	102
SupplyGraph	3	Convolution 1-D	LeakyReLU(alpha = 0.1)	(100, 220, 5)	1 × 5	1 × 1	100	110,100
	4	Convolution 1-D	LeakyReLU(alpha = 0.1)	(5, 100, 5)	1 × 5	1 × 1	5	2505

**Table 7:** Details of the edge classification part of the proposed method.

Data	Layer	Layer Name	Activation Function	Output Dimension
SupplyGraph (product-based connections) (4 categories)	1	Linear	ReLU	(Number of edges, 100)
	2	Linear	ReLU	(Number of edges, 4)
SupplyGraph (plant-based connections) (25 categories)	1	Linear	ReLU	(Number of edges, 100)
	2	Linear	ReLU	(Number of edges, 25)

**Table 8:** Details of training parameters.

Parameters	Search Scope	Optimal Value
Optimizer of graph section	Adam, SGD	Adam
Cost function of graph segment	MSE, cross-entropy	Cross-entropy
Number of Chebyshev convolutional layers	2, 3, 4	3
Learning rate of graph segment	0.1, 0.01, 0.001	0.001
Window size	15, 20, 25, 30	20
Optimizer of convolutional segment	Adam, SGD	Adam
Learning rate of convolutional segment	0.01, 0.001, 0.0001, 0.00001	0.0001
Number of convolutional layers of second segment	2, 3, 4	4

**Table 9:** Performance metrics of the proposed method (accuracy, precision, recall, F1-score).

DataCo Category	Ch-EGN (k_1_ = 1, k_2_ = 1, k_3_ = 1, k_4_ = 1)	Ch-EGN (k_1_ = 1, k_2_ = 2, k_3_ = 2, k_4_ = 2)	Ch-EGN (k_1_ = 2, k_2_ = 2, k_3_ = 2, k_4_ = 2)	Ch-EGN (k_1_ = 3, k_2_ = 3, k_3_ = 3, k_4_ = 3)	FCh-EGN	GAT-EGN	G-EGN
On-time delivery	98.2	98.3	94.7	93.2	95.3	93.8	91.8
Late delivery	99.7	97.8	95.2	92.6	94.9	93.5	91.1
Overall accuracy	98.95	98.05	94.95	92.9	95.1	93.65	91.45
Precision	99.7	97.81	95.2	93.2	94.9	93.50	91.74
F1-score	98.9	98.04	94.7	92.87	95.09	93.64	91.41
Recall	98.22	98.32	94.72	93.15	95.28	93.78	91.09

**Table 10:** Performance metrics of the proposed method for the SupplyGraph product classification scenario.

Supply Graph Categories	Ch-EGN	FCh-EGN	G-EGN	GAT-EGN
S	100	90.09	85.75	80.6
P	100	85.28	82.92	78.9
A	100	87.39	82.25	80.1
M	100	83.27	81.29	75.8
E	100	84.46	81.95	76.3
Overall accuracy	100	86.54	84.57	78.9
Precision	100	86.08	84.18	78.4
F1-score	100	86.03	84.11	78.3
Recall	100	86.09	84.2	78.5

**Table 11:** Accuracy for product relation classification.

Supply Graph Dataset	Ch-EGN (k_1_ = 1, k_2_ = 1, k_3_ = 1, k_4_ = 1)	Ch-EGN (k_1_ = 1, k_2_ = 2, k_3_ = 2, k_4_ = 2)	Ch-EGN (k_1_ = 2, k_2_ = 2, k_3_ = 2, k_4_ = 2)	Ch-EGN (k_1_ = 3, k_2_ = 3, k_3_ = 3, k_4_ = 3)	FCh-EGN	G-EGN	GAT-EGN
Product group classification (node classification)	100	100	100	100	86.54	84.57	80.09
Product group relation classification (edge classification)	98.07	98.07	96.1	94.47	85.2	81.54	79.32
Plant relation classification (edge classification)	92.37	92.37	90.03	88.68	82.3	80.76	78.44

**Table 12:** Comparison with the conventional methods.

Method	Product Group Classification	Product Group Relation Classification	Plant Relation Classification
Ch-EGN	100	98.07	92.37
GNN-based [47]	75.68	91.36	91.45
KNN [48]	64.44	74.75	74.63
XGB [49]	65.56	71.78	71.23
Logistic regression	66.67	62.73	68.63

## Data Availability

The datasets used in this study are publicly available at the following address links: https://www.kaggle.com/datasets/azminetoushikwasi/supplygraph-supply-chain-planning-using-gnns (accessed on 1 January 2024); https://www.kaggle.com/datasets/shashwatwork/dataco-smart-supply-chain-for-big-data-analysis (accessed on 1 January 2019).
